# A Radicular Cyst With Extensive Bone Loss Requires Surgical and Endodontic Management: Case Presentation and Literature Review

**DOI:** 10.1155/crid/9977128

**Published:** 2025-04-28

**Authors:** Juan Martín Pesántez Alvarado, Milton Fabricio Lafebre Carrasco

**Affiliations:** ^1^Department of Oral Surgery and Pathology, Dentistry Faculty, University of Cuenca, Cuenca, Ecuador; ^2^Department of Periodontics and Implantology, Dentistry Faculty, University of Cuenca, Cuenca, Ecuador

**Keywords:** endodontic, radicular cyst, surgical, treatment

## Abstract

The radicular cyst is an inflammatory pathology affecting the periapical bone tissue associated with teeth exhibiting pulp necrosis. Without timely endodontic intervention, this pathology can lead to significant bone tissue destruction due to its growth pattern, necessitating both endodontic and surgical approaches to halt its progression and remove the involved tissue. This case study details the management of a 23-year-old patient with a history of prior trauma, presenting with pressure in the anterior maxillary region. Tomographic examination revealed a hypodense area involving Teeth 2.1, 2.2, and 2.3. A surgical approach involving enucleation and endodontic treatment was undertaken. The diagnosis was confirmed histopathologically, and follow-up radiographs demonstrated satisfactory bone filling.

## 1. Introduction

Extensive carious lesions, defective restorations, iatrogenic orthodontic procedures, trauma, or parafunctional habits can precipitate an infectious or degenerative process in the root canals. This process initiates an immune response, potentially leading to granuloma formation, which attempts to repair or initially contain the local inflammatory process. Bone resorption is a severe complication, and over time, the epithelial remnants of Malassez proliferate, leading to a periapical cyst [1–4].

The epithelial lining of these cysts is predominantly stratified squamous, with a smaller percentage presenting respiratory-type epithelium (ciliated columnar cells). The prevalence of such lesions, as reported by Ricucci et al., ranges from 6% to 55%, with differential diagnosis from granulomas achieved histopathologically [1, 5].

Several theories have been proposed for the origin of radicular cysts. One theory is nutritional deficiency; epithelial cells proliferate uncontrollably and die due to the inflammatory stimulus, generating a cystic cavity [6]. Another theory posits that an abscess in the connective tissue is surrounded by epithelial cells, forming a cyst [1, 7]. A third theory involves epithelial proliferation, forming a three-dimensional structure that degenerates to create the cystic cavity [6].

Ricucci et al. classify cystic lesions based on their communication with the root canals into pocket cysts (direct communication) and true cysts (no communication) [1].

The pathogenesis of periapical cysts involves the immune system's response to bacteria such as *Enterococcus faecalis* and facultative anaerobes, including *Actinomyces israelii* and *Propionibacterium propionicum*. These microorganisms invade the dentinal tubules and pulp chamber, leading to necrosis and odontogenic infection [8]. The immune response activates inflammatory and mesenchymal cells to produce mediators such as prostaglandins, interleukins, proteases, and proinflammatory cytokines (TNF alpha, IL-1, and IL-6) that induce osteoclast formation and bone resorption. Bacterial endotoxins exacerbate cystic growth and bone destruction.

Radiographically, radicular cysts appear as radiolucent, rounded, or oval unilocular areas in the periapical region, often distinguished by a radio-opaque line associated with sclerotic bone. When the radiolucent area exceeds 1.6 cm and extends to the apices of neighboring teeth, an inflammatory radicular cyst is likely [9]. Differential diagnoses include periapical granuloma, bone scar, simple bone cyst, traumatic bone cyst, or keratinized odontogenic tumor [10, 11]. Diagnosis involves correlating clinical symptoms, pulp vitality tests, and radiographic findings, with histopathology providing definitive confirmation, in which fibrous connective tissue, granulation tissue, epithelium, and acute or chronic inflammatory cells are observed. Lymphocytes, plasmocytes, macrophages, and neutrophils predominate [2].

This work is aimed at presenting the clinical case of an extensive radicular cyst inflammatory disease, its clinical and radiographic characteristics, surgical treatment (enucleation), definitive diagnosis through histopathological study, and review of the literature on the pathological process.

## 2. Case Presentation

**Figure 1:**
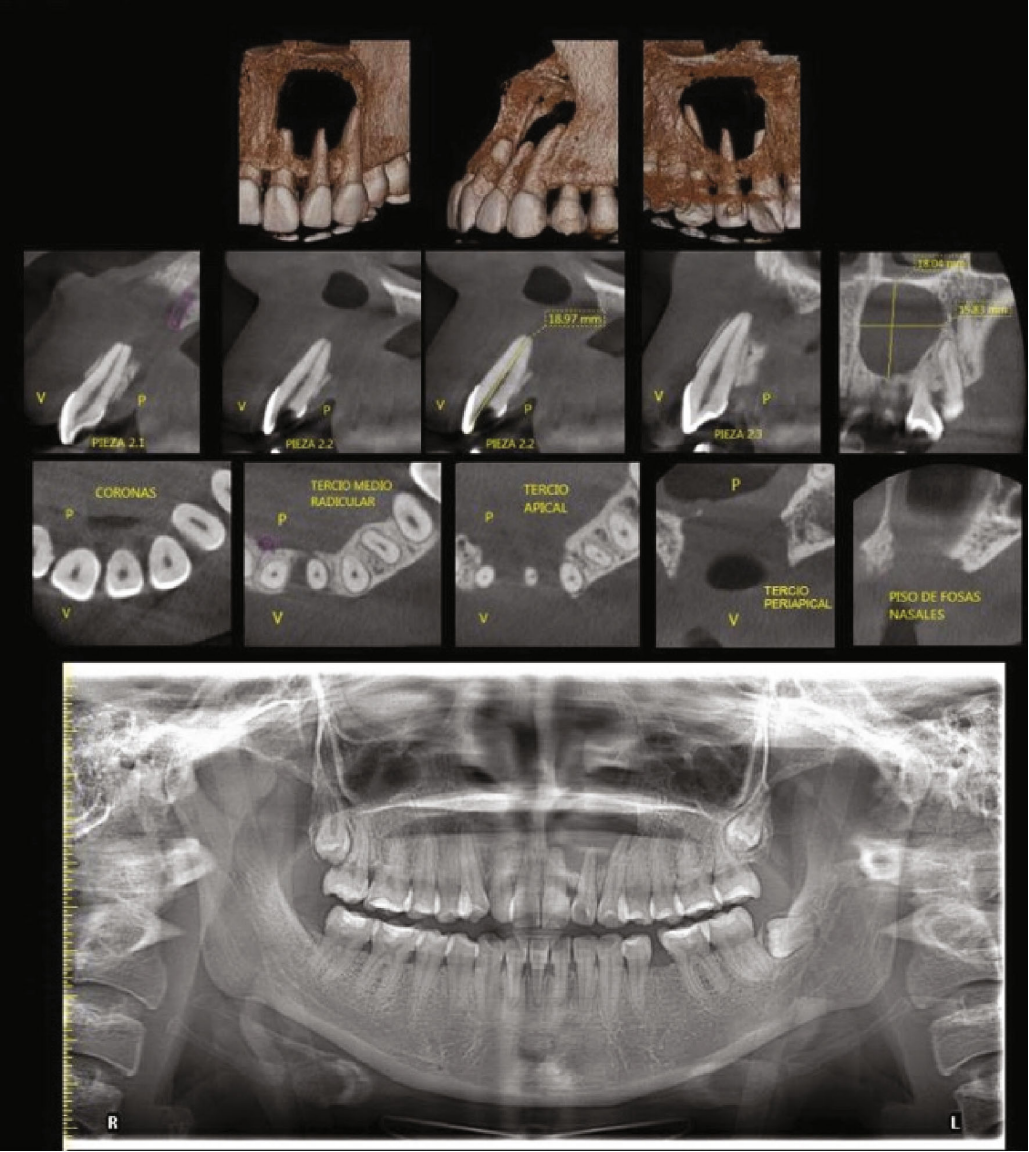
Initial CBCT study showing large osteolytic lesion.

A 23-year-old woman presented to a specialized consultation following a referral from general dentistry. She reported a pressure sensation in the anterior maxillary region, associated with a trauma 2 years prior. Clinical examination revealed Tooth 2.2 with a palatal cameral opening and purulent drainage. Initial panoramic radiographs showed a sizeable radiolucent area apical to Tooth 2.2, involving Teeth 2.1 and 2.3. Cone beam computed tomography (CBCT) revealed an 18 mm by 15 mm hypodense area, eroding the buccal and palatal bone tables (Figure 1).

**Figure 2:**
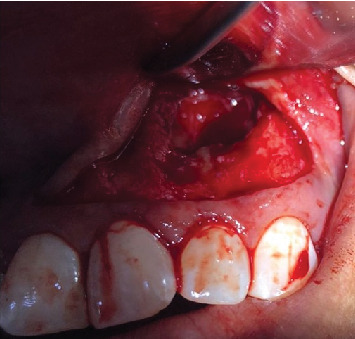
Surgical access to the lesion.

**Figure 3:**
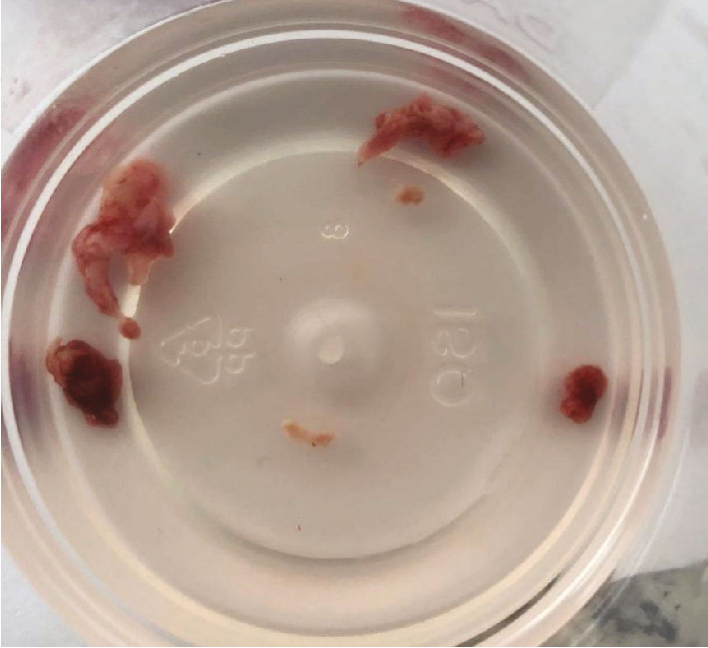
Sample obtained from the surgical procedure.

**Figure 4:**
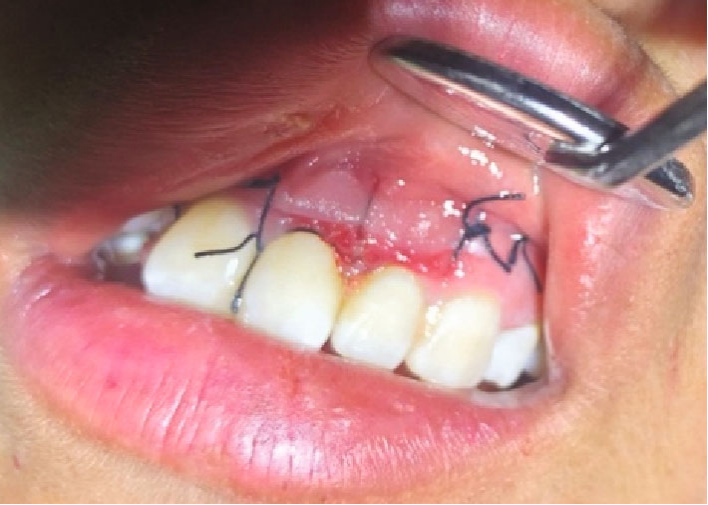
Postoperative control after 7 days.

After clinical and imaging analysis, a presumptive diagnosis of an inflammatory apical cyst was made. Given the lesion's extent, a surgical approach was chosen to decompress and clean the periapical area. A semilunar flap was raised, and the vestibular bone table was perforated for access (Figure 2), followed by enucleation, systematic curettage, and suturing. Due to patient's economic condition, no graft material was used. Subsequently, endodontic treatment is performed on Tooth 2.1 and 2.2, and no retrograde material filling was used. In addition, Tooth 2.3 is maintained with expectant management since the vitality tests are positive. Histopathological analysis confirmed the diagnosis (Figure 3), and follow-up radiographs showed adequate bone filling at 1 year (Figure 4).

**Figure 5:**
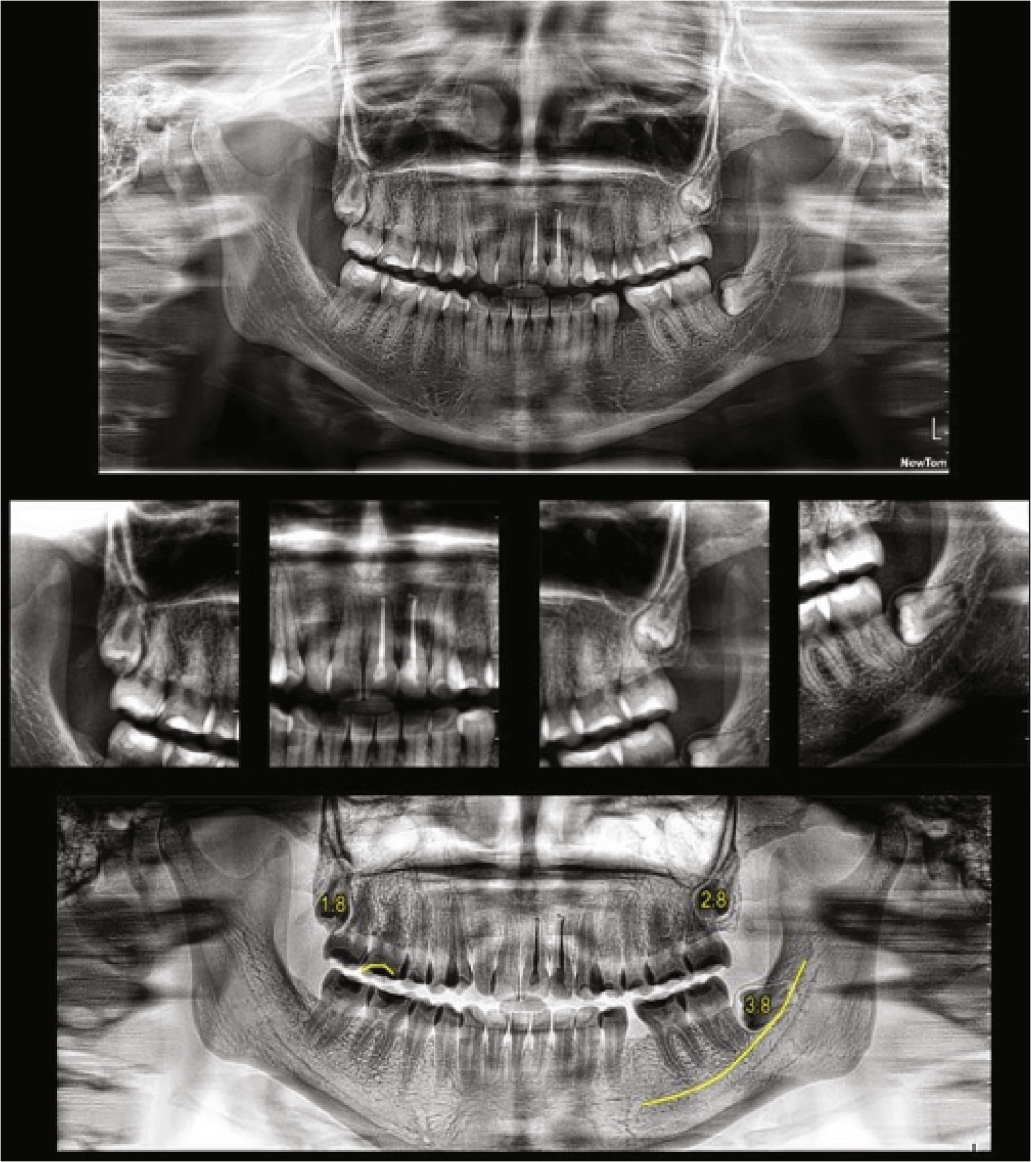
Annual radiographic control.

A control x-ray was performed after 1 year, observing adequate bone filling without the presence of the lesion (Figure 5).

**Figure 6:**
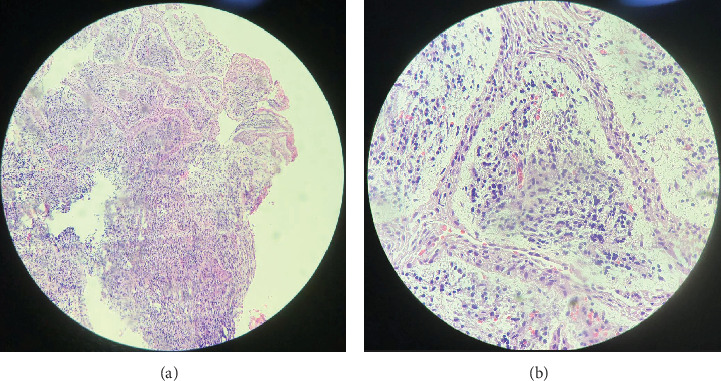
(a) Cystic lesion (4x). (b) Cystic epithelium surrounding predominantly chronic granulation tissue (40x).

**Figure 7:**
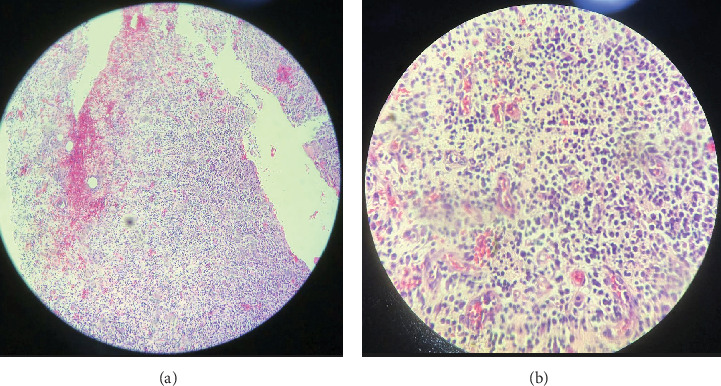
(a) Granulation tissue (4x). (b) Granulation tissue: vascular congestion and predominantly chronic inflammatory infiltrate (40x).

Histopathological examination revealed hyperplastic stratified squamous epithelium (Figure 6a,b), and granulation tissue with collagen fibers, hyperemic blood vessels, and a chronic inflammatory infiltrate (Figure 7a,b).

## 3. Discussion

This clinical case allowed us to diagnose a large radicular cyst, approximately 18 mm high by 15 mm wide, located apically to Teeth 2.1, 2.2, and 2.3. The cyst's development was attributed to the lack of a trauma management protocol, which should have included pulp vitality tests and periapical radiographs at 3 and 6 months post-trauma. The patient did not have access to dental services in her location, and as the painful symptoms subsided, she did not seek specialized consultations. Two years later, the patient presented with a sensation of pressure, and radiographic and clinical examinations confirmed the presence of the cyst.

The location of the lesion in this case aligns with findings reported by Lin et al., who analyzed 252 samples of inflammatory lesions (128 granulomas and 117 cysts) and found that 186 were located in the jaw, representing 68.4% of the sample [12]. Chen et al. reported an even higher percentage, with 86.6% of inflammatory lesions in the jaw. They also noted that the upper lateral incisor is the tooth most frequently affected by inflammatory lesions with 38.9% [13], corroborated by Love and Firth, who found a 35.8% frequency of inflammatory lesions in the upper lateral incisor in 100 cases [14]. These findings support the presumptive diagnosis in our case, as the characteristics described are consistent with the literature.

Another significant factor noted by Chen et al. is that the majority of cases in their study involved women between the third and fifth decades of life [13], a finding supported by Couto et al., who reported that 56.1% of 10,381 chronic inflammatory lesion cases in Brazil involved women with an average age of 37.01 years, with radicular cyst being the most common lesion [15]. Our patient, however, is younger than the typical age range, a variation likely due to the etiology of dentoalveolar trauma.

The diagnostic process for the lesion involved reviewing the patient's history, conventional x-ray, and CBCT imaging. These imaging modalities revealed tissue changes, but cellular-level changes necessitate a histopathological study for a definitive diagnosis. Radicular cyst diagnosis relies on clinical, imaging, and histopathological findings. The primary differential diagnosis is granuloma, distinguishable through histopathological examination. De Rosa et al. highlighted that CBCT can differentiate granulomas from radicular cysts based on the grayscale pixel levels representing different lesion components, aiding oral and maxillofacial radiologists in definitive diagnosis [16].

Histopathological characteristics in this case were consistent with Chen et al.'s findings that most cystic cavities are lined with nonkeratinized stratified squamous epithelium and predominantly chronic inflammatory infiltrate [13], correlating with the lesion's chronic development as indicated by the patient.

For large radicular cysts, surgical treatment options include decompression, marsupialization, and enucleation, chosen based on lesion size. Enucleation in large lesions can affect adjacent structures [17], so conservative approaches like decompression or marsupialization are preferred. These methods reduce lesion size by lowering hydrostatic pressure on bone tissue, allowing subsequent enucleation without risk to adjacent structures. In our case, enucleation was performed because the lesion had perforated the vestibular bone table, enabling complete removal while preserving nearby anatomical structures. Cho et al. demonstrated that decompression reduces lesion volume and enhances bone remodeling [17], a result mirrored in our case where enucleation relieved intraosseous pressure, promoting bone regeneration. Kunhappan et al. reported successful nonsurgical management of periapical lesions using triantibiotic paste (ciprofloxacin, metronidazole, and minocycline) and MTA apical root filling [18], a finding corroborated by Fernandes and de Ataide, who noted high success rates for nonsurgical endodontic treatments in teeth with inflammatory pathology. However, they emphasized the necessity of decompression when exudate is present [19]. In our case, purulent exudate observed during initial access justified the surgical approach for lesion management. Consequently, we chose a surgical approach to obtain a lesion sample for histopathological confirmation. Given the lesion's size, bone destruction, and impact on neighboring teeth evidenced by CBCT, conventional endodontic treatment followed, aligning with the management criteria for large cysts described by Ricucci et al. and Lin et al. [3, 12].

## 4. Conclusions

Radicular cysts are inflammatory pathologies of infectious origin that require prompt endodontic treatment. Delayed intervention can lead to significant lesion enlargement and intraosseous pressure, necessitating surgical management to eliminate the lesion and stimulate osteoblastic activity for tissue regeneration.

## Data Availability

The clinical data supporting the findings of this case report are contained within the patient's medical record, maintained at the corresponding author's private dental practice. Due to the sensitive nature of these data and in compliance with ethical standards and patient confidentiality regulations, they are not publicly accessible. Deidentified data may be made available from the corresponding author upon a justified request and pending appropriate institutional or ethical approval.
